# Diagnostic performance of Sonazoid-enhanced CEUS in identifying definitive hepatocellular carcinoma in cirrhotic patients according to KLCA-NCC 2022 and APASL 2017 guidelines

**DOI:** 10.1186/s13244-024-01838-x

**Published:** 2024-10-31

**Authors:** Zhe Huang, Rong-Hua Zhu, Shan-shan Li, Hong-Chang Luo, Kai-Yan Li

**Affiliations:** 1 Department of Medical Ultrasound, Tongji Hospital, Tongji Medical College, Huazhong University of Science and Technology, Wuhan, 430030 China; 2 Institute of Hepato-Pancreato-Biliary Surgery, Tongji Hospital, Tongji Medical College, Huazhong University of Science and Technology, Wuhan City, Hubei Province China

**Keywords:** Hepatocellular carcinoma, Contrast-enhanced ultrasound, Liver, Diagnostic imaging

## Abstract

**Objective:**

This study aims to assess the diagnostic performance of Sonazoid-contrast-enhanced ultrasound (CEUS) in identifying definitive HCC within hepatic nodules in cirrhotic patients, comparing the KLCA-NCC 2022 and APASL 2017 diagnostic guidelines.

**Materials and methods:**

This retrospective study analyzed cirrhotic patients who underwent Sonazoid-CEUS for liver lesion evaluation between October 2019 and October 2023. HCC diagnosis was based on the KLCA-NCC 2022 and APASL 2017 guidelines. Inter-reader agreement on CEUS imaging features and the diagnostic accuracy of the guidelines were evaluated. Sensitivity and specificity comparisons were made using McNemar’s test.

**Results:**

Among 400 patients with 432 lesions, CEUS showed excellent inter-reader consistency in detecting arterial phase hyperenhancement and Kupffer defects. The KLCA-NCC 2022 criteria notably enhanced sensitivity to 96.2%, with specificity and accuracy of 93.8% and 95.8%, respectively. APASL 2017 achieved the highest sensitivity at 97.8%, although specificity dropped to 46.9%, resulting in an accuracy of 90.3%. The KLCA-NCC 2022 guidelines demonstrated significantly higher specificity than APASL 2017 (*p* < 0.001), while APASL 2017 exhibited the highest sensitivity at 97.8%. Notably, the KLCA-NCC 2022 guidelines also demonstrated an impressive positive predictive value of 98.9%.

**Conclusion:**

Sonazoid-enhanced CEUS, particularly when applied using the KLCA-NCC 2022 guidelines, is an effective diagnostic tool for HCC.

**Critical relevance statement:**

Perfluorobutane CEUS, particularly in accordance with the KLCA-NCC 2022 guidelines, emerges as a valuable adjunct for diagnosing HCC in cirrhotic patients. It demonstrates superior positive predictive value and specificity compared to APASL 2017, underscoring its potential as an effective diagnostic tool.

**Key Points:**

- Contrast-enhanced (CE)US using Sonazoid with KLCA-NCC 2022 guidelines is highly effective for HCC diagnosis.
- KLCA-NCC 2022 criteria showed high accuracy, 96.2% sensitivity, and 98.9% PPV.
- CEUS demonstrated excellent inter-reader consistency in detecting arterial phase hyperenhancement and Kupffer defects.

**Graphical Abstract:**

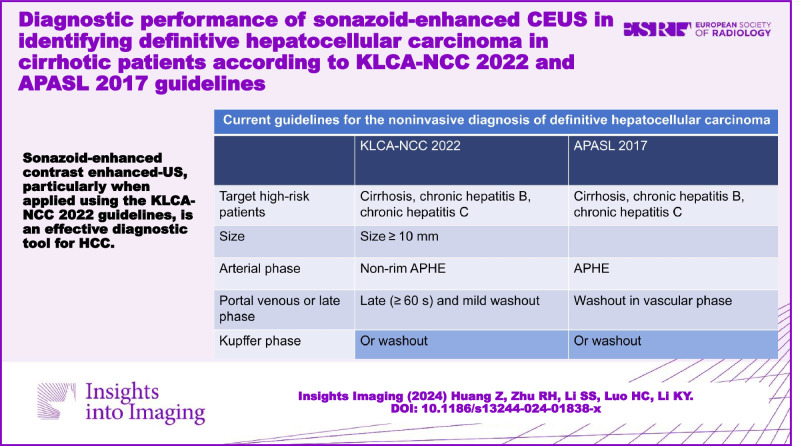

## Introduction

Liver cancer stands as the third leading cause of cancer-related mortality globally [1]. Among primary liver cancers, hepatocellular carcinoma (HCC) is the most common, accounting for 75% of such malignancies [2]. Cirrhosis is identified as a significant risk factor for HCC, originating from a variety of sources, including chronic viral hepatitis, excessive alcohol intake, both inherited and acquired metabolic diseases like NAFLD, hereditary hemochromatosis, and occasionally, alpha-1 antitrypsin deficiency. Statistically, one-third of individuals diagnosed with cirrhosis are at risk of developing HCC in their lifetime [3], with longitudinal studies indicating that 1–8% of cirrhosis patients annually progress to HCC [4].

The diagnosis of HCC can now be determined noninvasively, eliminating the need for histopathological confirmation, especially when characteristic imaging features are present in high-risk patients. A host of international guidelines, including those from the European Association for the Study of the Liver (EASL) [5], the endorsement by the American Association for the Study of Liver Diseases (AASLD) of the Liver Imaging Reporting and Data System (LI-RADS) algorithm [6, 7], the guidelines by the Korean Liver Cancer Association-National Cancer Center (KLCA-NCC) [8], and the Asia Pacific Association for the Study of the Liver (APASL) [9], provide criteria for HCC diagnosis. Initially, the utility of contrast-enhanced ultrasound (CEUS) was questioned due to the potential misdiagnosis of intrahepatic cholangiocarcinoma, which was considered a significant risk for misinterpretation [10, 11]. Subsequent research, however, has shown that the majority of intrahepatic cholangiocarcinoma cases exhibit a washout phenomenon within 60 s post-contrast injection—a pattern seen in 50–85% of cases and less commonly in HCC [12–14]. Furthermore, the washout intensity during the portal venous phase is significantly more pronounced in intrahepatic cholangiocarcinoma compared to HCC [15], facilitating more accurate identification of HCC’s typical markers in CEUS imagery. Considering these findings, the phrasing of certain national guidelines, including the EASL’s, has been revised. Nonetheless, a comprehensive comparison of the diagnostic accuracy of Sonazoid-CEUS for definitive HCC identification across various international guidelines is still lacking.

Therefore, this study aims to evaluate the diagnostic performance of Sonazoid-CEUS in identifying definitive HCC within hepatic nodules in cirrhotic patients, specifically comparing the effectiveness of the KLCA-NCC 2022 and APASL 2017 guidelines.

## Materials and methods

### Patients

**Fig. 1:**
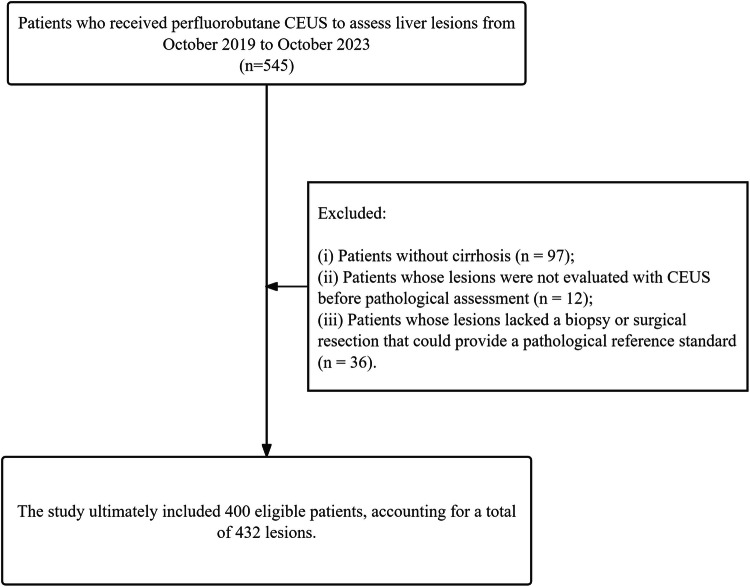
Flow chart of patient recruitment

This study, approved by the institutional review board which waived the need for written informed consent, conducted a retrospective analysis of patients who underwent perfluorobutane CEUS for liver lesion assessment between October 2019 and October 2023. The focus was strictly on primary HCC. After excluding patients without cirrhosis (*n* = 97), those whose lesions were not evaluated by CEUS before pathological assessment (*n* = 12), and those lacking a biopsy or surgical resection for a pathological reference (*n* = 36), 400 eligible patients and 432 lesions were included in the study (Fig. 1). Cirrhosis was confirmed based on histopathological results.

### Examination procedure of CEUS with Sonazoid

CEUS examinations were performed on a LOGIQ 9 US system (GE Healthcare, Wauwatosa, WI), using a transducer operating between 1 and 9 MHz. The procedures were conducted by a radiologist with 30 years of experience in grayscale ultrasound and over a decade in liver CEUS. Patients were advised to hold their breath or take shallow breaths during the examination for consistent imaging quality. Initially, a thorough conventional and color Doppler ultrasound assessment was performed for each patient, followed by the CEUS examination. This involved a single bolus injection of perfluorobutane (Sonazoid, 0.015 mL/kg, GE, Waukesha, WI, USA) intravenously, succeeded by a 10 mL flush of 0.9% saline solution through an antecubital vein. Upon contrast agent injection, the CEUS operating mode and a chronograph were simultaneously activated. The scanning phases included: the AP, starting with the arrival of microbubbles in the hepatic artery and ending as they filled the hepatic parenchyma; the early portal venous phase, within 1 min of injection; and the late portal venous phase, within 2 min of injection. The Kupffer phases commenced over 10 min post-injection. Imaging began immediately after contrast administration and continued for 15 min.

Two radiologists (Z.H. and S.S.L., with 6 and 10 years of post-training experience in abdominal imaging, respectively) independently reviewed the grayscale images and CEUS digital cine clips of all enrolled patients. The radiologists were blinded to the patients’ medical and surgical histories, laboratory results, CT and/or MRI findings, and pathological outcomes. For each observation, the two radiologists documented the following features: the pattern of arterial phase hyperenhancement (APHE), classified as either non-rim or rim, along with no APHE (hypoenhancement, isoenhancement, or peripheral globular enhancement). They also assessed the onset and degree of washout—categorized as no washout, early washout (< 60 s), marked washout (< 2 min), late-onset washout (≥ 60 s), or late and mild washout (≥ 60 s). Washout is defined by a visually assessed temporal reduction in enhancement in whole or in part relative to the composite liver tissue, resulting in hypoenhancement at any time [16]. Additionally, in the same session, they evaluated the presence of a Kupffer defect, which is characterized by hypoechogenicity (i.e., diminished enhancement) in the Kupffer phase compared to the surrounding liver parenchyma [17]. In cases of discrepancies between the two assessments, a third, more experienced radiologist with 35 years of experience in liver imaging was consulted to reassess the images until a consensus was reached.

### Diagnosis of HCC

**Fig. 2:**
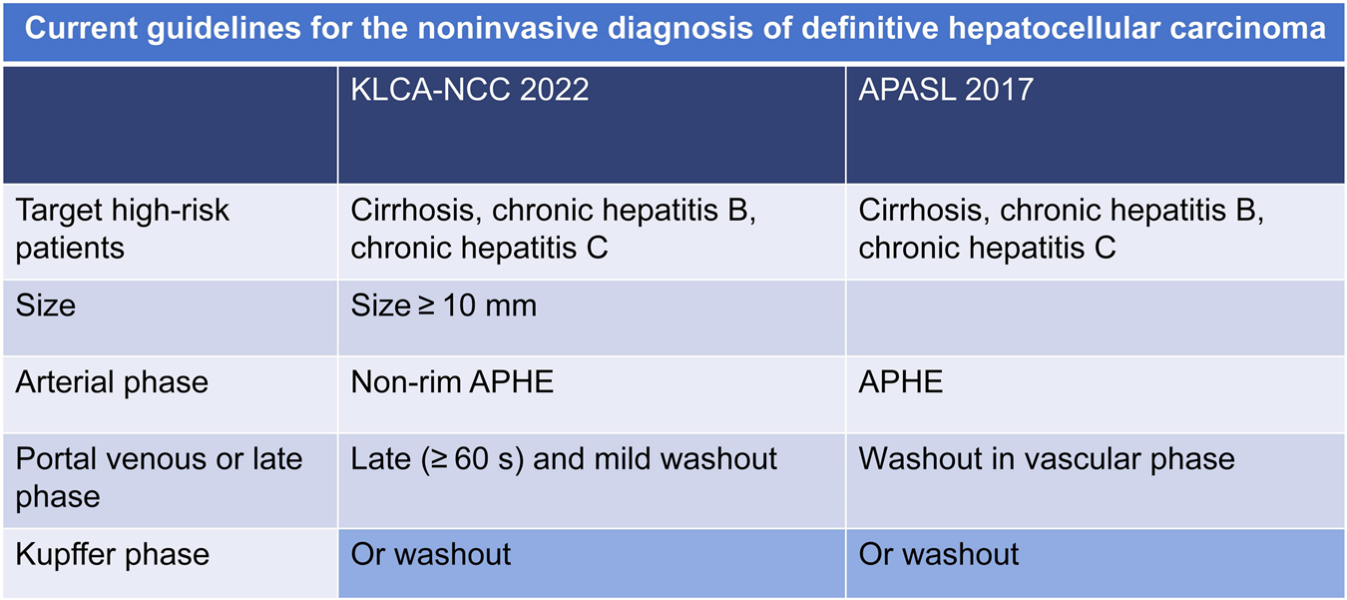
Current guidelines for the noninvasive diagnosis of definitive hepatocellular carcinoma. KLCA-NCC, Korean Liver Cancer Association-National Cancer Center; APASL, Asia Pacific Association for the Study of the Liver

The study employed various international guidelines for the definitive diagnosis of HCC on CEUS (Fig. 2).

KLCA-NCC 2022: Criteria specify lesions ≥ 10 mm with non-rim APHE and either late (≥ 60 s) and mild washout or washout in the Kupffer cell phase.

APASL 2017: Identifies HCC through APHE, washout in the vascular phase, or a hypoechoic appearance in the Kupffer cell phase, without a size limit.

### Histopathological analysis

All tissue samples were obtained from biopsy or routine therapeutic surgeries performed. After fixation in neutral buffered formalin, all tissue specimens were embedded in paraffin. According to a standard protocol, 4 μm thick tissue sections were cut and stained with hematoxylin and eosin (HE). Histological and immunohistochemical analyses were performed by two experienced liver pathologists. The histological grade of HCC was determined according to the World Health Organization (WHO) criteria [18]. HCCs were subdivided into three groups according to their degree of malignancy: G1, well-differentiated; G2, moderately differentiated; and G3, poorly differentiated. The diagnosis of angiomyolipoma required the identification of smooth muscle cells, adipose tissue, and blood vessels within the hepatic tissue, with immunohistochemical positivity for homatropine methylbromide–45. Epithelioid hemangioendothelioma was confirmed by the presence of endothelial markers (CD31, CD34, and factor VIII-related antigen (FVIII Ag)) in the tissue, indicating endothelial origin.

### Statistical analysis

Quantitative data were presented as mean ± standard deviation or median with interquartile range, contingent on their distribution. Categorical variables were depicted as counts and percentages. The degree of inter-reader agreement was evaluated using κ value, categorized as follows: 0.00–0.20 indicating slight agreement; 0.21–0.40, fair; 0.41–0.60, moderate; 0.61–0.80, good; and 0.81–1.00, excellent. Diagnostic performance metrics such as sensitivity, specificity, positive and negative predictive values, and overall accuracy were calculated. Sensitivity and specificity comparisons were made using McNemar’s test. All statistical analyses were conducted using SPSS Statistics 22.0 (IBM, Armonk, USA), with a *p*-value of less than 0.05 deemed statistically significant.

## Results

### Patient cohort

The comprehensive details of the clinical and pathological characteristics of the participants in this study are meticulously detailed in Table 1. The cohort consisted of 400 patients (62 females), ranging in age from 13 to 82 years. Pathological diagnoses were confirmed via surgical resection in 401 cases and biopsy in 31 cases. Among these, 368 were HCC, 33 were non-HCC malignant lesions, and 31 with benign lesions.

**Table 1:** The clinicopathological features of patients

Clinicopathological features	Patients (*n* = 400)
Male, *n* (%)	338 (84.5)
Age, mean ± SD, years	54 (47–64)
Cirrhosis, *n* (%)	
Hepatitis B	328 (82.0)
Non-alcoholic steatohepatitis	21 (5.3)
Hepatitis C	2 (0.5)
Alcohol-related liver disease	46 (11.5)
Other	3 (0.8)
AFP, ng/mL	18.5 (4.3–438.2)
CEA, ng/mL	2.3 (1.6–3.8)
CA125, ng/mL	10.0 (7.8–14.8)
Alanine aminotransferase, U/L	25.0 (17.0–37.8)
Aspartate aminotransferase, U/L	27.0 (20.2–36.0)
Total bilirubin, μmol/L	12.1 (8.8–15.4)
Pathology, *n* (%)	Lesions (*n* = 432)
HCC	368 (85.2)
G1	159 (39.8)
G2	64 (16.0)
G3	145 (36.3)
Intrahepatic cholangiocarcinoma	22 (5.1)
Combined hepatocellular-cholangiocarcinoma	4 (0.9)
Epithelioid hemangioendothelioma	1 (0.3)
Leiomyosarcoma	1 (0.3)
Carcinosarcoma	2 (0.5)
Adenocarcinoma	1 (0.3)
Metastases	2 (0.5)
Hemangioma	11 (2.5)
Focal nodular hyperplasia	16 (3.7)
Angiomyolipoma	1 (0.3)
Hepatocellular adenoma	1 (0.3)
Regenerative nodules or dysplastic nodules	2 (0.5)

*AFP* alphafetoprotein, *CEA* carcinoembryonic antigen, *CA125* carbohydrate antigen125

The 33 non-HCC malignant lesions encompassed 22 cases of intrahepatic cholangiocarcinoma, 4 cases of combined hepatocellular-cholangiocarcinoma, 1 case of epithelioid hemangioendothelioma, 1 case of leiomyosarcoma, 2 cases of carcinosarcoma, 1 case of adenocarcinoma, and 2 cases of metastases. Out of the 31 benign lesions, there were 11 hemangiomas, 16 instances of focal nodular hyperplasia, 1 angiomyolipoma, 1 hepatocellular adenoma, and 2 cases of regenerative or dysplastic nodules.

### CEUS features

Table 2 summarizes the inter-reader agreement on imaging characteristics. The findings show good agreement on arterial phase hyperenhancement, with κ values ranging from 0.775 to 0.793. Similarly, during the portal venous and late phases, the agreement ranged from good to excellent (κ values between 0.753 and 0.838). Notably, the presence of Kupffer defects was identified with excellent agreement, underscored by a κ value of 0.876.

**Table 2:** Inter-reader agreement for imaging findings

Characteristics	κ (95% CI)
Arterial phase	
Non-rim APHE	0.775 (0.721–0.833)
Rim APHE	0.793 (0.714–0.867)
Portal venous or late phase	
No washout	0.838 (0.732–0.965)
Early washout (< 60 s)	0.786 (0.717–0.853)
Late-onset washout (≥ 60 s)	0.753 (0.705–0.786)
Late and mild washout (≥ 60 s)	0.794 (0.718–0.861)
Marked washout (< 2 min)	0.814 (0.727–0.896)
Kupffer phase	
Presence of Kupffer defect	0.876 (0.758–0.974)

*APHE* arterial phase hyperenhancement

Table 3 details the observed imaging characteristics. Within the 368 HCC lesions, a remarkable 99.7% (367/368) exhibited non-rim arterial phase hyperenhancement (APHE) on perfluorobutane CEUS, and 78.9% (287/368) demonstrated late and mild washout. Moreover, 97.8% (360/368) of HCC showed a Kupffer defect. Additionally, the perfluorobutane CEUS identified a Kupffer defect in 57.8% (37/64) of non-HCC lesions. Washout characteristics: Early washout, Late-onset washout, Late and mild washout, Marked washout, and Presence of Kupffer defect show significant differences between HCC and non-HCC (*p* < 0.001).

**Table 3:** Comparison of frequency of imaging features among groups of observations

Characteristics	HCC (*n* = 368)	Non-HCC (*n* = 64)
Echogenicity (hyper, hypo)	100:268	47:17
Tumor size (cm)	4.1 (2.7–6.5)	4.7 (2.5–7.6)
Blood flow signal (yes, no)	270 (73.4)	40 (62.5)
Arterial phase		
No APHE	1 (0.3)	2 (3.1)
APHE	367 (99.7)	62 (96.9)
Non-rim APHE	367 (99.7)	46 (71.9)
Rim APHE	0 (0)	16 (25.0)
Portal venous or late phase		
No washout	44 (12.0)	28 (43.8)
Washout	324 (88.0)	36 (56.3)
Early washout (< 60 s)	37 (10.1)	28 (43.8)
Late-onset washout (≥ 60 s)	287 (78.9)	8 (12.5)
Late and mild washout (≥ 60 s)	287 (78.9)	4 (6.3)
Marked washout (< 2 min)	0 (0)	4 (6.3)
Kupffer phase		
Presence of Kupffer defect	360 (97.8)	37 (57.8)

*APHE* arterial phase hyperenhancement

### Diagnostic accuracy of different modalities

Table 4 outlines the diagnostic efficacy of CEUS for HCC across different guideline criteria. The KLCA-NCC 2022 criteria notably enhanced sensitivity to 96.2%, with a specificity and accuracy of 93.8% and 95.8%, respectively. The APASL 2017 criteria topped sensitivity at 97.8%, although specificity dipped to 46.9%, resulting in an accuracy of 90.3%.

**Table 4:** Diagnostic performance of various guidelines for definitive hepatocellular carcinoma identification

Guidelines	Sensitivity	Specificity	Positive predictive value	Negative predictive value	Accuracy
KLCA-NCC 2022	0.962 [0.936, 0.978]	0.938 [0.840, 0.980]	0.989 [0.970, 0.996]	0.811 [0.670, 0.889]	0.958 [0.914, 0.983]
APASL 2017	0.978 [0.956, 0.990]	0.469 [0.345, 0.597]	0.914 [0.880, 0.939]	0.789 [0.622, 0.899]	0.903 [0.864, 0.933]

*KLCA-NCC* Korean Liver Cancer Association-National Cancer Center, *APASL* Asia Pacific Association for the Study of the Liver

**Fig. 3:**
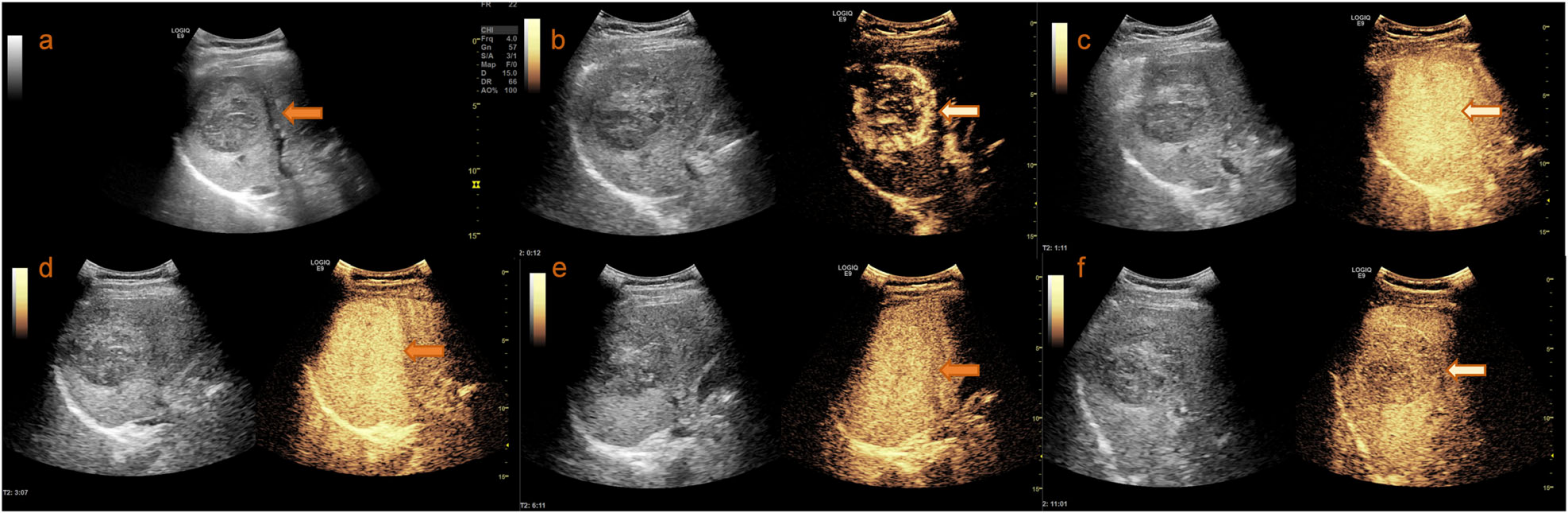
Imaging and diagnosis of HCC. **a** Identification of a liver mass located in the right lobe, discovered during a routine physical examination. **b** Enhancement of the mass following the injection of contrast agent in the arterial phase and portal venous or late phase. **c**–**f** Kupffer phase imaging shows early washout of the contrast agent from the mass. Therefore, this lesion could be accurately diagnosed as HCC under the KLCA-NCC and APASL guidelines

The KLCA-NCC 2022 guidelines demonstrated the highest specificity, significantly surpassing that of APASL 2017 (*p* < 0.001). Conversely, APASL 2017 exhibited superior sensitivity at 97.8%, notably exceeding KLCA-NCC 2022 (*p* < 0.001). Furthermore, the KLCA-NCC 2022 guidelines achieved an impressive positive predictive value of 98.9% (Fig. 3).

## Discussion

The landscape of HCC diagnosis has significantly shifted toward noninvasive methodologies, obviating the need for histopathological evidence, especially when specific imaging signatures are present in patients with a high-risk profile. As the refinement of CEUS diagnostic criteria continues, CEUS may present a valuable evaluation tool. The need to further explore the diagnostic accuracy of Sonazoid-CEUS for the definitive identification of HCC under diverse international guidelines grows more pressing. Our study has revealed that the KLCA-NCC 2022 guidelines demonstrated significantly higher specificity than APASL 2017. In contrast, APASL 2017 exhibited the highest sensitivity at 97.8%. Impressively, KLCA-NCC 2022 guidelines also demonstrated a remarkable positive predictive value of 98.9%.

Although CEUS is widely used in Europe and North America for liver imaging, it does not offer a complete view. The short duration of the arterial phase with these contrast agents is insufficient for a comprehensive scan of the entire liver, and it may be difficult to detect deep-seated lesions. Despite this limitation, CEUS remains an indispensable alternative in specific clinical scenarios. It is particularly valuable when CT/MRI imaging modalities are contraindicated, such as for young patients with chronic liver disease who are at risk from CT radiation, when MRI is unsuitable due to incompatible devices or induces claustrophobia, or when patients have allergies to conventional contrast agents or suffer from renal insufficiency.

In a prospective multicenter study, Sonovue CEUS showed a decrease in sensitivity only for nodules measuring 10–20 mm and exhibited the highest sensitivity and specificity for nodules in the 20–30 mm range when compared to CT/MRI [19]. The lower sensitivity of Sonovue CEUS for HCC has been attributed to a lower rate of washout [5]. In this study, the KLCA-NCC 2022 and APASL 2017 guidelines displayed comparable sensitivity, potentially due to the inclusion of Kupffer defect assessment with perfluorobutane, which enhances the sensitivity of ultrasound contrast imaging for HCC. Notably, only 88.0% of HCCs demonstrated a washout pattern during the portal venous or late phase of CEUS, while a notably higher proportion, 97.8%, showed defects during the Kupffer phase. The findings indicate the potential impact of selecting perfluorobutane as the contrast agent for CEUS, to allow lesion characterization in the Kupffer phase.

To maintain a high specificity for the diagnosis of HCC, noninvasive diagnostic criteria are reserved for patients with significant risk factors. The KLCA-NCC and the APASL guidelines also consider patients with cirrhosis, chronic hepatitis B, and chronic hepatitis C to be at high risk for HCC [8, 9]. Therefore, this study has exclusively selected patients with cirrhosis to align with the study scopes of both guidelines.

This study has several limitations. First, it is based on a single-center and is retrospective in design, which may introduce potential confounders due to selection bias. The requirement for a pathological reference standard also contributes to selection bias, affecting the prevalence of various diagnoses in the study sample. Future studies with a larger and more balanced sample of liver lesions are necessary to validate our findings under less biased conditions. Moreover, the study cohort was limited to patients with cirrhosis. Consequently, it is not feasible to extrapolate our results to non-cirrhotic patients. While CEUS offers high diagnostic accuracy for characterizing focal liver lesions, it will never replace MRI or CT. The inherent limitations of the technique, such as challenges in imaging obese patients or those with significant ascites, stem from its physical properties.

In summary, Sonazoid-CEUS has proven to be a valuable diagnostic instrument for the diagnosis of HCC in patients with cirrhosis. This is particularly evident when employing the criteria set forth by the KLCA-NCC 2022 guidelines, which have demonstrated remarkable specificity and positive predictive value.

## Data Availability

The dataset used or analyzed during the current study are available from the corresponding author upon reasonable request.

## Abbreviations

APASL: Asia Pacific Association for the Study of the Liver
CEUS: Contrast-enhanced ultrasound
HCC: Hepatocellular carcinoma
KLCA-NCC: Korean Liver Cancer Association-National Cancer Center

## References

[1] Sung H, Ferlay J, Siegel RL et al (2021) Global cancer statistics 2020: GLOBOCAN estimates of incidence and mortality worldwide for 36 cancers in 185 countries. CA Cancer J Clin 71:209–249 33538338 10.3322/caac.21660

[2] McGlynn KA, Petrick JL, El-Serag HB (2021) Epidemiology of hepatocellular carcinoma. Hepatology 73:4–13 32319693 10.1002/hep.31288 PMC7577946

[3] Sangiovanni A, Prati GM, Fasani P et al (2006) The natural history of compensated cirrhosis due to hepatitis C virus: a 17-year cohort study of 214 patients. Hepatology 43:1303–1310 16729298 10.1002/hep.21176

[4] Ioannou GN, Splan MF, Weiss NS, McDonald GB, Beretta L, Lee SP (2007) Incidence and predictors of hepatocellular carcinoma in patients with cirrhosis. Clin Gastroenterol Hepatol 5:938–945 17509946 10.1016/j.cgh.2007.02.039

[5] European Association for the Study of the Liver (2018) EASL clinical practice guidelines: management of hepatocellular carcinoma. J Hepatol 69:182–236 10.1016/j.jhep.2018.03.019 29628281

[6] Singal AG, Llovet JM, Yarchoan M et al (2023) AASLD practice guidance on prevention, diagnosis, and treatment of hepatocellular carcinoma. Hepatology 78:1922–1965 37199193 10.1097/HEP.0000000000000466 PMC10663390

[7] Marrero JA, Kulik LM, Sirlin CB et al (2018) Diagnosis, staging, and management of hepatocellular carcinoma: 2018 practice guidance by the American Association for the Study of Liver Diseases. Hepatology 68:723–750 29624699 10.1002/hep.29913

[8] Korean Liver Cancer Association (KLCA), National Cancer Center (NCC) Korea (2022) 2022 KLCA-NCC Korea practice guidelines for the management of hepatocellular carcinoma. Clin Mol Hepatol 28:583–705 10.3350/cmh.2022.0294 PMC9597235 36263666

[9] Omata M, Cheng AL, Kokudo N et al (2017) Asia-Pacific clinical practice guidelines on the management of hepatocellular carcinoma: a 2017 update. Hepatol Int 11:317–370 28620797 10.1007/s12072-017-9799-9 PMC5491694

[10] Vilana R, Forner A, Bianchi L et al (2010) Intrahepatic peripheral cholangiocarcinoma in cirrhosis patients may display a vascular pattern similar to hepatocellular carcinoma on contrast-enhanced ultrasound. Hepatology 51:2020–2029 20512990 10.1002/hep.23600

[11] Terzi E, Iavarone M, Pompili M et al (2018) Contrast ultrasound LI-RADS LR-5 identifies hepatocellular carcinoma in cirrhosis in a multicenter restropective study of 1006 nodules. J Hepatol 68:485–492 29133247 10.1016/j.jhep.2017.11.007

[12] Li R, Zhang X, Ma KS et al (2013) Dynamic enhancing vascular pattern of intrahepatic peripheral cholangiocarcinoma on contrast-enhanced ultrasound: the influence of chronic hepatitis and cirrhosis. Abdom Imaging 38:112–119 22323003 10.1007/s00261-012-9854-x

[13] Yuan MX, Li R, Zhang XH et al (2016) Factors affecting the enhancement patterns of intrahepatic cholangiocarcinoma (ICC) on contrast-enhanced ultrasound (CEUS) and their pathological correlations in patients with a single lesion. Ultraschall Med 37:609–618 25919414 10.1055/s-0034-1399485

[14] Liu GJ, Wang W, Lu MD et al (2015) Contrast-enhanced ultrasound for the characterization of hepatocellular carcinoma and intrahepatic cholangiocarcinoma. Liver Cancer 4:241–252 26779444 10.1159/000367738 PMC4702012

[15] Wildner D, Pfeifer L, Goertz RS et al (2014) Dynamic contrast-enhanced ultrasound (DCE-US) for the characterization of hepatocellular carcinoma and cholangiocellular carcinoma. Ultraschall Med 35:522–527 25202903 10.1055/s-0034-1385170

[16] Kim DH, Choi JI (2021) Current status of image-based surveillance in hepatocellular carcinoma. Ultrasonography 40:45–56 33045812 10.14366/usg.20067 PMC7758104

[17] Lee JY, Minami Y, Choi BI et al (2020) The AFSUMB consensus statements and recommendations for the clinical practice of contrast-enhanced ultrasound using Sonazoid. Ultrasonography 39:191–220 32447876 10.14366/usg.20057 PMC7315291

[18] Fletcher CDM, Unni KK, Mertens F (2002) Pathology and genetics of tumours of soft tissue and bone. In: Christopher DM, Unni K, Krishnan K (eds) World Health Organization; International Agency for Research on Cancer. Lyon, IARC Press

[19] Aubé C, Oberti F, Lonjon J et al (2017) EASL and AASLD recommendations for the diagnosis of HCC to the test of daily practice. Liver Int 37:1515–1525 28346737 10.1111/liv.13429

